# Qigong for Hypertension: A Systematic Review

**DOI:** 10.1097/MD.0000000000000352

**Published:** 2015-01-09

**Authors:** Xingjiang Xiong, Pengqian Wang, Xiaoke Li, Yuqing Zhang

**Affiliations:** From the Department of Cardiology, Guang’anmen Hospital, China Academy of Chinese Medical Sciences (XX); Bio-organic and Natural Products Laboratory, McLean Hospital, Harvard Medical School, Belmont, MA, USA (XL); Institute of Basic Research in Clinical Medicine, China Academy of Chinese Medical Sciences (PW), Beijing, China; Bio-organic and Natural Products Laboratory, McLean Hospital, Harvard Medical School, Belmont, MA; and Department of Clinical Epidemiology and Biostatistics, McMaster University, Hamilton, ON, Canada (YZ).

## Abstract

The purpose of this review was to evaluate the efficacy and safety of qigong for hypertension.

A systematic literature search was performed in 7 databases from their respective inceptions until April 2014, including the Cochrane Library, EMBASE, PubMed, Chinese Scientific Journal Database, Chinese Biomedical Literature Database, Wanfang database, and Chinese National Knowledge Infrastructure. Randomized controlled trials of qigong as either monotherapy or adjunctive therapy with antihypertensive drugs versus no intervention, exercise, or antihypertensive drugs for hypertension were identified. The risk of bias was assessed using the tool described in *Cochrane Handbook for Systematic Review of Interventions*, version 5.1.0.

Twenty trials containing 2349 hypertensive patients were included in the meta-analysis. The risk of bias was generally high. Compared with no intervention, qigong significantly reduced systolic blood pressure (SBP) (weighted mean difference [WMD] = −17.40 mm Hg, 95% confidence interval [CI] −21.06 to −13.74, *P* < 0.00001) and diastolic blood pressure (DBP) (WMD = −10.15 mm Hg, 95% CI −13.99 to −6.30, *P* < 0.00001). Qigong was inferior to exercise in decreasing SBP (WMD = 6.51 mm Hg, 95% CI 2.81 to 10.21, *P* *=* 0.0006), but no significant difference between the effects of qigong and exercise on DBP (WMD = 0.67 mm Hg, 95% CI −1.39 to 2.73, *P* *=* 0.52) was identified. Compared with antihypertensive drugs, qigong produced a clinically meaningful but not statistically significant reduction in SBP (WMD = −7.91 mm Hg, 95% CI −16.81 to 1.00, *P* *=* 0.08), but appeared to be more effective in lowering DBP (WMD = −6.08 mm Hg, 95% CI −9.58 to −2.58, *P* *=* 0.0007). Qigong plus antihypertensive drugs significantly lowered both SBP (WMD = −11.99 mm Hg, 95% CI −15.59 to −8.39, *P* < 0.00001) and DBP (WMD = −5.28 mm Hg, 95% CI, −8.13 to −2.42, *P* = 0.0003) compared with antihypertensive drugs alone. No serious adverse events were reported.

The meta-analysis suggests that qigong is an effective therapy for hypertension. However, more rigorously designed randomized controlled trials with long-term follow-up focusing on hard clinical outcomes are required to confirm the results.

## INTRODUCTION

Hypertension is a common condition in the general population that remains one of the most important risk factors for cardiovascular disease and stroke worldwide. For patients not currently undergoing treatment with antihypertensive agents, hypertension is typically characterized by systolic blood pressure (SBP) ≥ 140 mm Hg and/or diastolic blood pressure (DBP) ≥ 90 mm Hg on at least 2 separate intervals (after a 4-week washout period).^1^ Blood pressure (BP) control in hypertensive patients is an effective intervention for reducing hypertension-associated cardiovascular and renal complications. Achieving BP control in hypertensive patients often requires multiple medications and trial-and-error switching of drug classes.^2^ Hundreds of compounds representing multiple drug classes were developed nearly 50 years ago. The guidelines on hypertension released by the Eighth Joint National Committee (JNC 8) reflect an evidence-based approach in recommending a therapeutic regimen that includes a healthy diet, weight control, regular exercise, and appropriate initial and supplemental antihypertensive therapy.^3^ There has been a revival of interest in complementary and alternative medicine (CAM) approaches for lowering BP ^4–6^ in part because control rates for hypertension remain dismal despite the vast armamentarium of antihypertensive agents available to clinicians.^3^ In some cases, hypertensive patients can benefit from drug therapy optimization and combination therapy. In some patients, however, adequate BP control cannot be achieved by these approaches.^7^ Another major impetus for current clinical trials using CAM as a therapeutic strategy is the many adverse effects of long-term conventional antihypertensive drugs.^8^ Therefore, some traditional therapies, including qigong,^9^ tai chi,^10^ acupuncture,^11^ moxibustion,^12^ yoga,^13^ massage,^14^ dietary supplements,^15^ and herbal products,^16,17^ are increasingly favored by hypertensive patients with the hope of lowering BP and relieving hypertension-related symptoms with fewer adverse effects.

Qigong is an ancient Chinese healing art that dates back 7000 years.^18^ The word “qigong” involves 2 theories: “qi,” the vital energy of the body, and “gong,” the training or cultivation of qi.^19^ The practice of qigong aims to cultivate energy via systematic training exercises, including the coordination of different breathing patterns, rhythmic movements, and meditation, in contrast to conventional exercise.^20^ Due to its significant promotion of human health and ease of learning, qigong is appropriate for nearly anyone of any age or physical condition. Qigong has its underpinnings in traditional Chinese medicine (TCM) and philosophy. One possible explanation for the beneficial effects of qigong exercise is increased healthy flow of qi, blood, and fluid throughout the body by repetitive movements to relieve pathological stagnation and regulate the function of meridians and visceral organs. Qigong is practiced by 5% of China's 1.3 billion people to improve health, explore the latent ability of humans, prevent disease, and prolong life in the context of a wide range of conditions, including hypertension,^21,22^ heart failure,^23^ coronary heart disease,^24^ cardiac rehabilitation,^25^ cancer,^26^ diabetes,^27^ metabolic syndrome,^28^ chronic obstructive pulmonary disease,^29^ movement disorders,^30^ chronic pain,^31^ fatigue,^32^ stress,^33^ anxiety,^34^ depression,^35^ and immune function^36^ and to enhance the quality of life (QOL) of patients with other chronic diseases.^37^

There is increasing evidence that qigong can benefit hypertensive patients, and clinical studies and systematic reviews of the efficacy of qigong as a useful lifestyle intervention for hypertension are ongoing. Qigong has been reported to decrease BP smoothly and moderately, recover the circadian rhythm of BP, improve QOL, slow the progression of severe complications, and reduce mortality.^9,21,22,38–40^ Until now, positive evidence of BP reduction by qigong has been summarized in 2 systematic reviews published in English based on the poor quality of the primary data.^21,22^ However, due to different study designs, 4 main databases in Chinese were not included in the retrieval randomized controlled trials (RCTs); thus, these 2 systematic reviews are subject to insufficient literature retrieval.^41^ For health practitioners, the appropriateness of recommending qigong as an effective therapy for hypertension remains unclear. This study aims to minimize bias and provide more-reliable findings by using explicit and systematic methods. To determine whether qigong safely benefits hypertensive patients, we performed a systematic review and meta-analysis of RCTs of qigong for hypertension.

## METHODS

This systematic review was conducted in accordance with the Preferred Reporting Items for Systematic Reviews and Meta-Analyses guidelines. Ethical approval was not necessary for this review study.

## ELIGIBILITY CRITERIA

### Types of Studies

Only RCTs evaluating the effects of qigong on hypertension were considered. Animal experiments were not included.

### Types of Participants

Participants of any sex, age, and ethnic origin were clinically diagnosed as hypertensive in any stage according to at least 1 of the current or past guidelines or definitions of hypertension. Trials that declared patients with hypertension but without detailed information about the diagnostic criteria were also considered.

### Types of Interventions

Eligible RCTs of qigong practiced alone or in combination with antihypertensive drugs versus no intervention, exercise, or antihypertensive drugs for hypertension were identified without restriction of blinding, publication status, or language. We excluded trials if interventions in the qigong or control groups contained other nonconventional therapies, including herbal medicine, acupuncture, moxibustion, cupping, and massage; studies reported only laboratory values rather than BP outcomes; or studies were case reports, case series, and duplicated publications reporting the same group of participants.

### Types of Outcome Measures

All trials used categorical or continuous BP as the outcome measure. The efficacy of qigong on categorical BP was evaluated using 3 graded criteria, as authoritatively recommended by the China Food and Drug Administration (available at http://www.sda.gov.cn) and the China National Committee on Screening, and Prevention of Coronary Heart Disease and High Blood Pressure of 1974.^22,42^ The evaluation criteria were as follows: “significant improvement” (DBP decreased by 10 mm Hg reaching the normal range or DBP not yet returned to normal but reduced by 20 mm Hg or more), “improvement” (DBP decreased by <10 mm Hg but reaching the normal range, DBP decreased by 10 to 19 mm Hg but not reaching the normal range, or SBP decreased by 30 mm Hg or more), and “no improvement” (not reaching the above standards). To permit the overall synthesis of these enumeration data, we grouped these data dichotomously, “significant improvement” and “improvement” as “effective” and “no improvement” as “ineffective.”

### Search Strategy

A systematic search was conducted in the following 7 online electronic databases from their inception until April 17, 2014: Cochrane Library (1996–2014), EMBASE (1980–2014), PubMed (1959–2014), Chinese Scientific Journal Database (1989–2014), Chinese Biomedical Literature Database (1978–2014), Wanfang database (1985–2014), and Chinese National Knowledge Infrastructure (1979–2014). We also reviewed the reference lists of retrieved articles. The websites of the Chinese Clinical Trial Registry (http://www.chictr.org/) and the international clinical trial registry of the US National Institutes of Health (http://clinicaltrials.gov/) were also searched to identify unpublished clinical trials. The following keywords were used to search the databases: (“hypertension” OR “high blood pressure” OR “blood pressure”) AND (“qigong” OR “qi gong” OR “chi gong” OR “chi kung”) AND (“clinical trial” OR “randomized controlled trial” OR “randomised controlled trial”).

### Study Selection

Two reviewers independently identified the potential literature and selected studies in a standardized manner. Then, the titles and abstracts of the references were screened for potentially relevant RCTs. The full texts of eligible articles were retrieved for further identification according to the specified selection criteria. Disagreements were resolved by consultation or consensus with a third reviewer.

### Data Extraction

Two reviewers independently extracted the following data: general information: authors, title, publication data, and literature source; characteristics of the included trials and patients: randomization, allocation concealment, blinding, intention-to-treat analysis, dropouts or withdrawals, sample size, age, sex, diagnosis standard, interventions of each group, and duration of treatment; outcomes: BP data at the baseline and after treatment; length and frequency of follow-up; and adverse events (AEs). Missing information was obtained from the original authors whenever possible.

### Quality Assessment

Two reviewers independently assessed the risk of bias in each included trial using Cochrane Collaboration's tool from the *Cochrane Handbook for Systematic Review of Interventions*, version 5.1.0.^43^ The following 7 aspects were evaluated: random sequence generation (selection bias), allocation concealment (selection bias), blinding of participants and personnel (performance bias), blinding of outcome assessment (detection bias), incomplete outcome data (attrition bias), selective reporting (reporting bias), and other bias (from Chapter 8: assessing risk of bias in included studies). Each item was categorized as low/high/unclear risk of bias. Then, the trials were categorized into 3 levels: low risk of bias (all items were at low risk of bias), high risk of bias (at least 1 item was at high risk for bias), and unclear risk of bias (at least 1 item was at unclear risk of bias).

### Data Synthesis

The Review Manager software (RevMan, version 5.1; The Nordic Cochrane Centre, The Cochrane Collaboration, Copenhagen, 2011) was used for data synthesis and analysis. Both dichotomous and continuous outcomes were extracted from the primary trials without any conversion. Dichotomous data were presented as risk ratio and continuous outcomes as weighted mean difference (WMD), both with a 95% confidence interval (CI). A meta-analysis was performed if the outcomes were similar in clinical characteristics. Heterogeneity between trials was recognized as significant when *I*^*2*^ > 50% or *P* *<* 0.1. The fixed-effects model was used to analyze data with low heterogeneity (heterogeneity test, *P* ≥ 0.10), whereas the random-effects model was applied if heterogeneity was significant (heterogeneity test, *P* < 0.10). Subgroup analysis was conducted according to the types of comparisons. Publication bias was assessed by funnel plot analysis if the group included >10 trials.^43^

## RESULTS

### Study Identification

**FIGURE 1:**
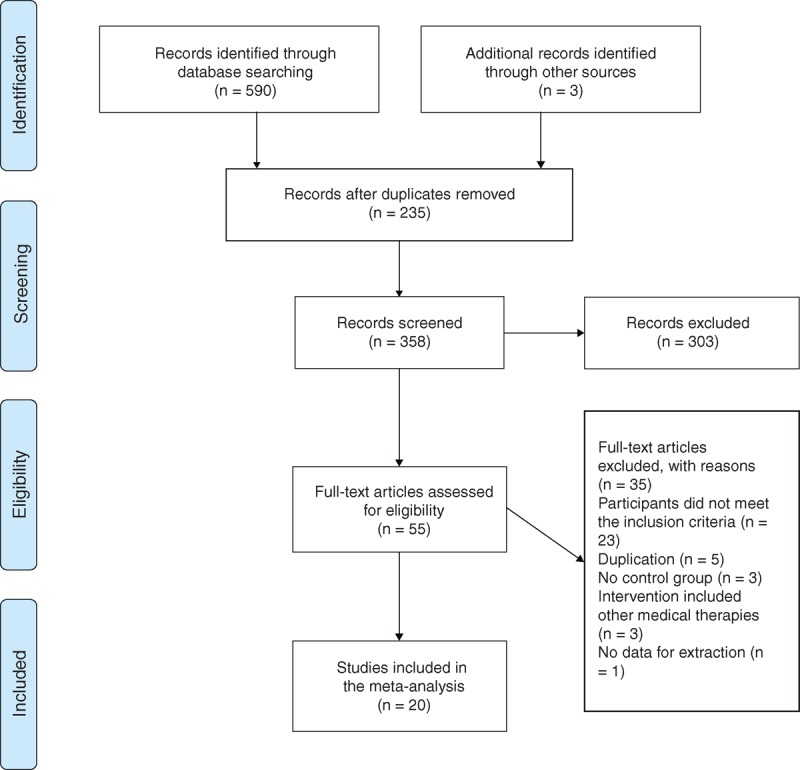
Flow diagram of study selection and identification.

We identified 593 articles on qigong for hypertension from electronic and manual searches. By reading the titles and abstracts, we excluded 235 duplicates of the articles that were included in different databases and 303 articles that were clearly review articles, experts’ commentaries, case reports, case series, or other nonclinical studies. A total of 55 full-text articles were retrieved for further assessment, of which 35 were excluded for the following reasons: participants did not meet the inclusion criteria (n = 23); duplication (n = 5); no control group (n = 3); intervention included other medical therapies, such as acupuncture, moxibustion, massage, and external application (n = 3); and no data for extraction (n = 1). Ultimately, 20 studies were included in this review.^44–63^ All trials were conducted in 2 countries (China, n = 18; South Korea, n = 2) and were published between 1982 and 2013 in 2 languages (Chinese, n = 17; English, n = 3). The flow diagram of the search and identification process is presented in Figure 1.

### Study Characteristics

A total of 2349 hypertensive patients were analyzed. All studies adopted qigong as either monotherapy or adjunctive therapy to antihypertensive drugs in the treatment group. Two trials ^47,53^ had a 3-group study design: 1 trial included qigong, jogging, and antihypertensive drugs groups,^47^ whereas the other included qigong plus antihypertensive drugs (QPADs), qigong, and antihypertensive drugs groups.^53^

Internal qigong (*neiqi*) and external qigong (*waiqi*) are distinguished by who is practicing qigong. That is, internal qigong (*neiqi*) is practiced by hypertensive patients themselves for beneficial health effects, whereas external qigong (*waiqi*) is performed by a trained practitioner other than the patient to direct the qi energy to the patient. In our review, all trials used internal qigong as the interventions in the treatment group. Interventions included qigong practiced alone^44–52^ and combined with antihypertensive drugs.^53–63^ The controls included wait-list control,^44,45^ conventional exercises,^46^ jogging,^47^ no exercise (maintenance of original lifestyle),^48^ oryzanol,^49^ or antihypertensive drugs.^50–63^ Patients in the treatment group received the same type and dosage of antihypertensive drugs under the same standard used for the control group. All studies were single-centered and parallel-designed.

The 20 included trials specified 3 diagnostic criteria of hypertension. Among these trials, 3 used the National Committee on Screening, and Prevention of Coronary Heart Disease and High Blood Pressure in China—1974,^47,52,62^ 3 trials used the National Committee on Prevention and Treatment of Cardiovascular Disease in China—1979,^53,54,60^ 2 trials used the World Health Organization-International Society of Hypertension guidelines for the management of hypertension guidelines for the management of hypertension—1978,^51,63^ and 12 trials included patients with hypertension without detailed information about diagnostic criteria.^44–46,48–50,55–59,61^

The total treatment duration ranged from 8 weeks to 12 months. Clinical efficacy on BP was observed in all studies, with no difference in the baseline BP data. Two different methods were used to evaluate the clinical efficacy on BP: 13 trials used BP data,^44–48,50–56,63^ whereas the other 7 trials used the 3-grade evaluation criteria.^49,57–62^ AEs were reported in only 1 trial,^46^ whereas no other studies mentioned them. The detailed study characteristics are summarized in Table 1 .

**TABLE 1:**
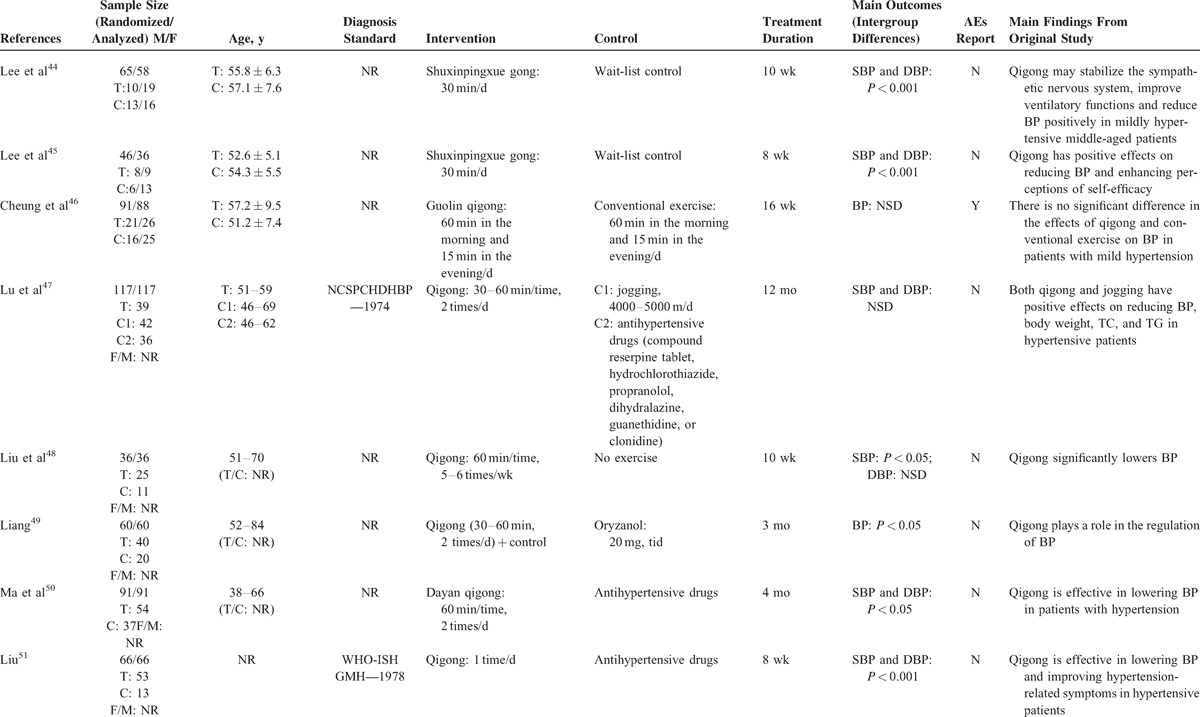
Basic Characteristics of the Included Studies

**TABLE 1 (Continued):**
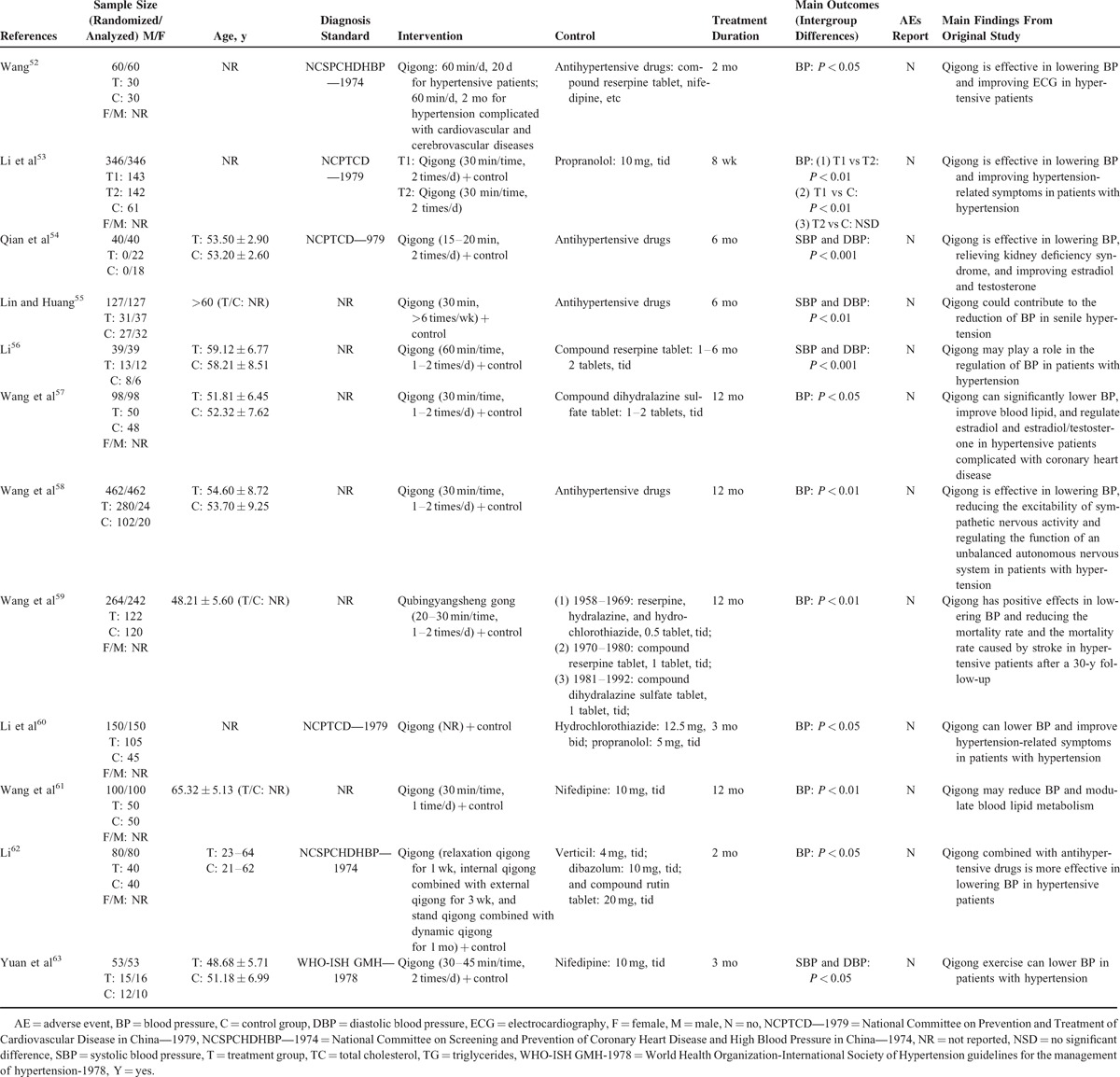
Basic Characteristics of the Included Studies

### Study Quality

We assessed the risk of bias of the included studies based on the information extracted from the methods section. According to the Cochrane Collaboration tool, most of the included trials were evaluated as high risk of bias. The methodological quality of most of the trials was generally “poor.” Only 1 study described the method for random sequence generation.^46^ No trials described allocation concealment, blinding of participants, or outcome assessment. Detailed information about dropouts and withdrawals were reported in 4 trials.^44–46,59^ Only 1 trial described a pretrial estimation of sample size.^46^ We also attempted to contact authors by telephone, fax, and email for further information, but no responses were received. The details of the risk of bias of each trial are presented in Table 2.

**TABLE 2:**
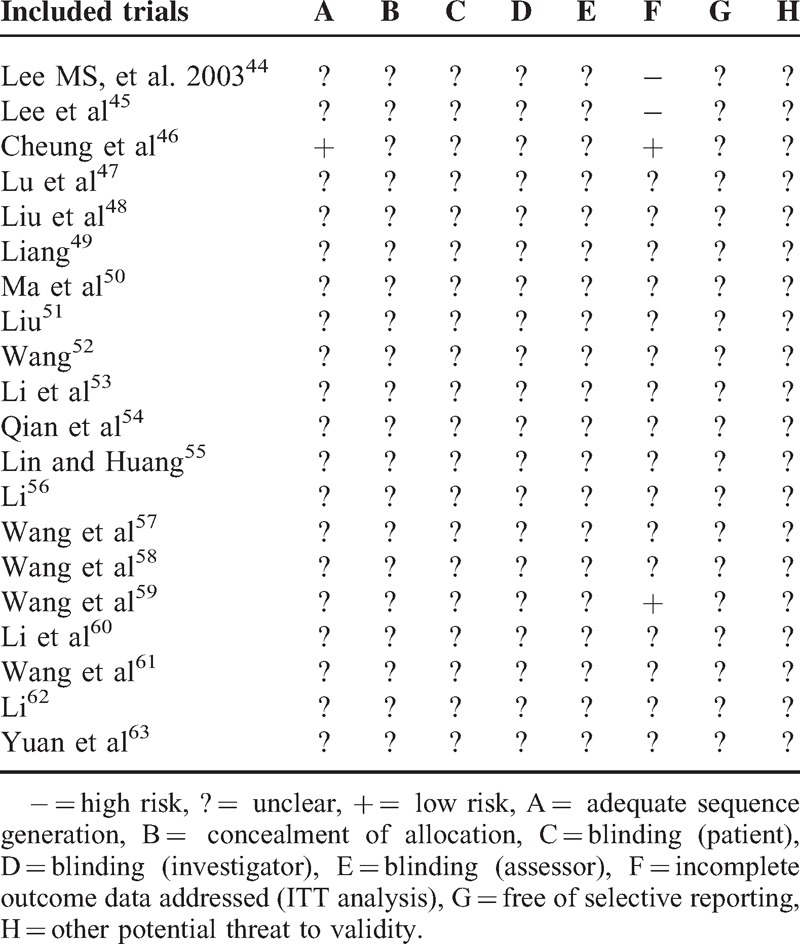
Methodological Quality of the Included Studies Based on the Cochrane Handbook

### Efficacy Assessment

All studies focused on the effect of qigong on hypertensive patients. A subgroup analysis was conducted among 4 different types of comparisons, including qigong versus no intervention,^44,45,48,49^ qigong versus exercise,^46,47^ qigong versus antihypertensive drugs,^47,50–53^ and QPADs versus antihypertensive drugs.^53–63^

#### Qigong Versus No Intervention

Interventions in the control groups of 4 trials included wait-list control,^44,45^ no exercise (maintenance of original lifestyle),^48^ and oryzanol.^49^ Because none of the interventions had an effect on BP, we grouped them into a “no-intervention” category for further analysis. There were 111 patients in the qigong group and 79 in the no-intervention group.

**FIGURE 2:**
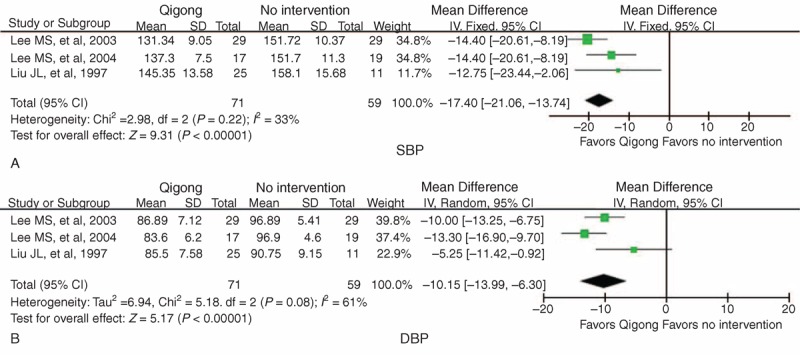
Forest plot of the comparison of qigong versus no intervention for the outcome of BP: (A) SBP and (B) DBP. BP = blood pressure, CI = confidence interval, DBP = diastolic blood pressure, SBP = systolic blood pressure.

Three trials used BP data to measure the clinical efficacy of qigong compared with no intervention.^44,45,48^ An overall synthesis of BP data was performed. Meta-analysis revealed a significant lowering effect of qigong on SBP (WMD = −17.40 mm Hg, 95% CI −21.06 to −13.74, *P* < 0.00001) with no significant heterogeneity (χ^2^ = 2.98, *P* = 0.22, *I*^2^ *=* 33%) (Figure 2A). Qigong significantly lowered the level of DBP (WMD = −10.15 mm Hg, 95% CI −13.99 to −6.30, *P* < 0.00001) with high heterogeneity (χ^2^ = 5.18, *P* *=* 0.08, *I*^2^ = 61%) compared with no intervention (Figure 2B).

One trial conducted by Liang^49^ used the 3-grade evaluation criteria to assess the effect of qigong on hypertension. A total of 60 hypertensive patients were randomized into a treatment group (n = 40) and control group (n = 20). The participants in the control group received oryzanol therapy, whereas the patients in the treatment group received qigong plus oryzanol therapy. After 3 months, the qigong plus oryzanol group had significantly lower BP in general than the oryzanol group (95% vs 35%, *P* < 0.01).

#### Qigong Versus Exercise

**FIGURE 3:**
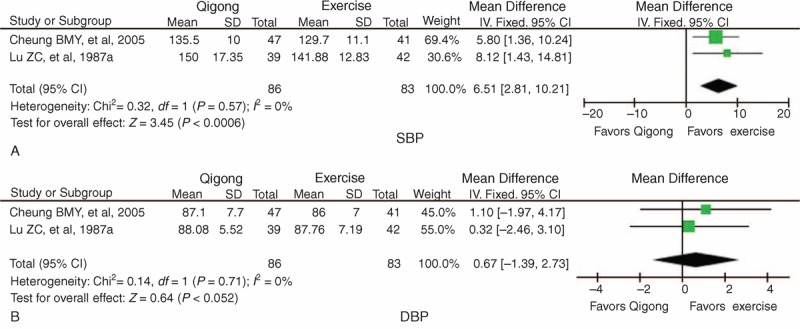
Forest plot of the comparison of qigong versus exercise for the outcome of BP: (A) SBP and (B) DBP. BP = blood pressure, CI = confidence interval, DBP = diastolic blood pressure, SBP = systolic blood pressure.

Because interventions in the control groups of 2 trials included conventional exercise^46^ and jogging,^47^ subgroup analysis of qigong versus exercise was performed. There were 86 patients in the qigong group and 83 in the exercise group. All studies reported BP data at baseline and after intervention. The meta-analysis indicated that SBP was significantly higher in the qigong group compared the exercise group after treatment (WMD = 6.51 mm Hg, 95% CI 2.81 to 10.21, *P* *=* 0.0006) with no significant heterogeneity (χ^2^ = 0.32, *P* = 0.57, *I*^2^ *=* 0%) (Figure 3A). There was no statistical significance in DBP between the qigong group and exercise group (WMD = 0.67 mm Hg, 95% CI −1.39 to 2.73, *P* *=* 0.52) with no significant heterogeneity (χ^2^ = 0.14, *P* = 0.71, *I*^2^ *=* 0%) (Figure 3B).

#### Qigong Versus Antihypertensive Drugs

Five studies evaluated the effect of qigong on BP compared with antihypertensive drugs.^47,50–53^ There were 318 patients in the qigong group and 177 in the antihypertensive drug group.

One trial conducted by Wang^52^ evaluated the effect of qigong on BP compared with antihypertensive drugs in 4 different age groups. The trial demonstrated a significant lowering effect of qigong on SBP and DBP. However, we could not use these data for further meta-analysis.

**FIGURE 4:**
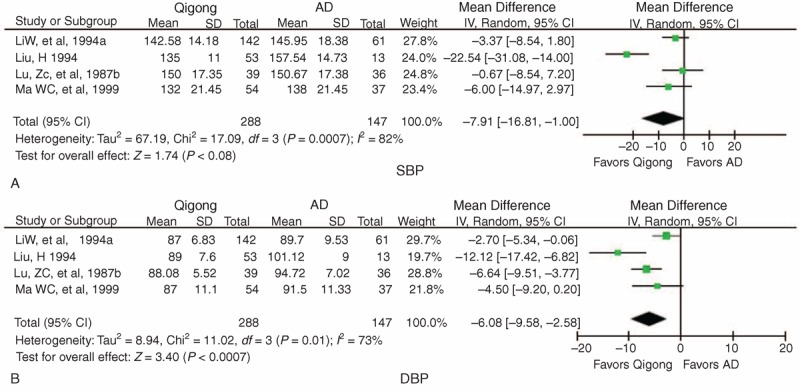
Forest plot of the comparison of qigong versus AD for the outcome of BP: (A) SBP and (B) DBP. AD = antihypertensive drugs, BP = blood pressure, CI = confidence interval, DBP = diastolic blood pressure, SBP = systolic blood pressure.

A change in BP data was also reported in 4 other trials,^47,50,51,53^ and subgroup analysis was performed. The meta-analysis did not reveal a significant lowering effect on SBP in the qigong group compared with the antihypertensive drug group (WMD = −7.91 mm Hg, 95% CI −16.81 to 1.00, *P* *=* 0.08) with high heterogeneity (χ^2^ = 17.09, *P* *=* 0.0007, *I*^2^ = 82%) (Figure 4A). Qigong significantly reduced DBP (WMD = −6.08 mm Hg, 95% CI −9.58 to −2.58, *P* *=* 0.0007) with high heterogeneity (χ^2^ = 11.02, *P* *=* 0.01, *I*^2^ = 73%) compared with antihypertensive drugs (Figure 4B).

#### QPADs Versus Antihypertensive Drugs

Eleven trials compared QPADs (combination therapy group) with antihypertensive drugs (single-therapy group) for the treatment of hypertension.^53–63^ There were 960 patients in the combination therapy group and 599 in the single-therapy group.

**FIGURE 5:**
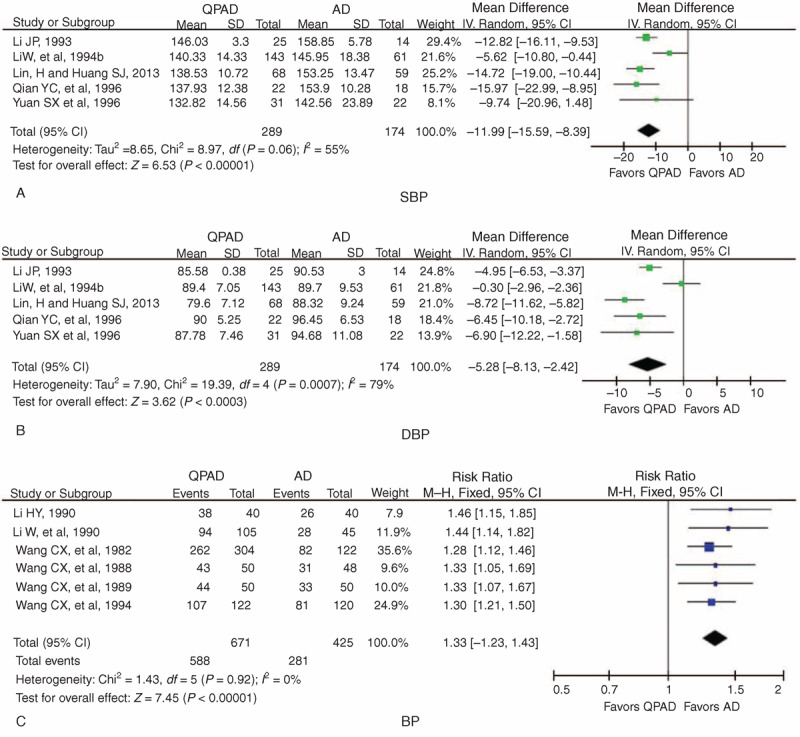
Forest plot of the comparison of QPAD versus AD for the outcome of BP: (A) SBP, (B) DBP, and (C) BP. AD = antihypertensive drugs, BP = blood pressure, CI = confidence interval, DBP = diastolic blood pressure, QPAD = qigong plus antihypertensive drugs, SBP = systolic blood pressure.

Five trials used BP data to evaluate the effect of combination therapy compared with single therapy,^53–56,63^ and subgroup analysis was performed. The pooled analysis of 5 trials indicated that compared with the single-therapy group, SBP was significantly lower in the combination therapy group (WMD = −11.99 mm Hg, 95% CI −15.59 to −8.39, *P* < 0.00001) with significant heterogeneity (χ^2^ = 8.97, *P* = 0.06, *I*^2^ *=* 55%) (Figure 5A). A significant lowering effect on DBP was observed in the combination therapy group (WMD = −5.28 mm Hg, 95% CI −8.13 to −2.42, *P* = 0.0003), with significant heterogeneity (χ^2^ = 19.39, *P* *=* 0.0007, *I*^2^ *=* 79%) compared with the single-therapy group (Figure 5B).

Six trials used the 3-grade criteria to assess the effect of combination therapy on BP in general.^57–62^ A meta-analysis revealed a significant BP-lowering effect in the combination therapy group (risk ratio = 1.33, 95% CI 1.23 to 1.43, *P* < 0.00001) with no significant heterogeneity (χ^2^ = 1.43, *P* = 0.92, *I*^2^ *=* 0%) compared with the single-therapy group (Figure 5C).

### Adverse Events

Among the 20 included trials, only 1 (5%, 1/20) mentioned AEs.^46^ In that trial, a woman in the qigong group experienced vestibular neuronitis; however, this symptom was not related to the practice of qigong. Although AEs were not rigorously monitored and reported in the other 19 trials (95%, 19/20), most of the original authors declared that qigong was a relatively safe approach for various conditions, including hypertension. No severe AEs were reported.

### Publication Bias

Because the number of included trials in each subgroup was <10, funnel plots could not be applied to detect potential publication bias.

## DISCUSSION

### Summary of Evidence

CAM, including TCM, is increasing in popularity among the general population in Western countries.^64–67^ Hypertensive patients have joined this global trend ^68,69^ and often seek complementary therapies to conventional antihypertensive treatment without their physicians’ knowledge.^70^ Previous reports have estimated that CAM therapies are used by 29% to 69.5% of hypertensive patients.^71–73^ Among the most common forms of CAM therapies, qigong is one of the easiest and most accessible modes worldwide.^74,75^ In China, qigong has been practiced for its health-enhancing qualities for thousands of years. Qigong was also recently confirmed to improve aspects of the psychosocial, physical, and physiological domains in hypertensive patients. These benefits include relieving tinnitus,^76^ improving fatigue,^77,78^ alleviating depression,^79^ reducing stress and anxiety,^34^ and enhancing QOL,^80^ specifically targeting older adults.^81,82^ However, the selection of such therapies should be based on scientific studies and evidence-based medicine rather than patient preference.^83^

Regarding the role of qigong in the treatment of hypertension, we aimed to provide a systematic review and meta-analysis of both the English and Chinese literature to address the efficacy and safety of qigong in the treatment of hypertension. Although qigong is mainly practiced and researched in China, only a few Chinese databases were searched in the 2 previous systematic reviews,^21,22^ potentially excluding relevant studies and inducing selection bias.^41^ A prominent characteristic of this review was the extensive, unbiased literature search in various Chinese databases, which included much information about qigong that is not available in the English literatures. Although 12 and 9 trials were included in Lee et al's study^21^ in 2007 and Guo et al's study^22^ in 2008, respectively, the current review includes additional recently published clinical trials, potentially providing more convincing evidence to support the use of qigong for hypertension. This is the most comprehensive assessment of qigong for hypertension and provides new evidence for clinical professionals.

The overall results of the 20 included randomized trials with 2349 hypertensive patients suggest that qigong is superior to no intervention in lowering SBP (decreased by 17.40 mm Hg) and DBP (decreased by 10.15 mm Hg). Qigong is inferior to exercise in decreasing SBP (increased by 6.51 mm Hg), but no significant difference between qigong and exercise was observed for DBP (increased by 0.67 mm Hg). Qigong resulted in a clinically meaningful but not statistically significant reduction in SBP (decreased by 7.91 mm Hg) but appeared to be more effective in lowering DBP (decreased by 6.08 mm Hg) compared with antihypertensive drugs. As an adjunctive therapy to antihypertensive drugs, qigong significantly lowered SBP (decreased by 11.99 mm Hg) and DBP (decreased by 5.28 mm Hg) compared with antihypertensive drugs alone.

Previous studies conducted by Lewington et al^84^ suggested that each increase of 20 mm Hg in SBP or 10 mm Hg in DBP doubles the risk of cardiovascular and stroke events in individuals ages 40 to 69 years with a BP > 115/75 mm Hg. However, antihypertensive treatment substantially reduces this cardiovascular risk. In this review, one of the critical issues was the effectiveness of qigong in lowering BP. The BP-lowering effect of qigong was evaluated in 4 subgroups according to different comparisons. The first finding was evidence about the efficacy of qigong compared with the no-intervention control. Our meta-analysis suggested that qigong has beneficial effects for lowering both SBP and DBP compared with no intervention. This result is consistent with previously published meta-analyses,^21,22^ indicating that regular qigong practice may have positive effects for hypertensive patients.

Another valuable finding of this review was the comparison between qigong and exercise. Guidelines for the management of hypertension recommend exercise as the frontline strategy for key preventive lifestyle modification to reduce the risk of hypertension and to manage high BP.^1,6,85^ Hypertensive patients are advised to perform 30 to 60 minutes of moderate-to-vigorous–intensity aerobic exercise 4 to 7 days per week in addition to the activities of daily living. Numerous observational epidemiological studies, clinical trials, and meta-analyses have been conducted to summarize the growing numbers of trials addressing the effects of exercise on BP.^86–89^ These studies have demonstrated that acute and chronic aerobic exercise can contribute to reducing resting and ambulatory BP in hypertensive patients. In this review, a meta-analysis of 2 trials reporting adequate data revealed that exercise is superior to qigong in lowering SBP, and a relatively small but not significant lowering effect was observed for DBP, indicating that regular physical exercise maybe more effective than qigong in the management of hypertension. Similar results were observed in a previously published systematic review.^22^ However, due to the small sample size, short-term duration, and limited trials, the current conclusions should be treated with caution. In addition, previous national surveys in the United States have revealed that exercise is practiced by only 26% of hypertension patients and that patients >75 years of age are least likely to participate.^90^ In China, qigong is widely practiced by middle-aged and elderly people, some of whom are intolerant of moderate-to-vigorous–intensity aerobic exercise. Therefore, qigong may be more suitable for elderly patients as a gentle alternative to intense physical activities, particularly static qigong, which has low physical demands, although further evidence is needed.^91,92^

The third interesting finding of this review was the comparison between qigong and antihypertensive drugs. There is robust evidence from RCTs that a BP reduction of 10 mm Hg systolic or 5 mm Hg diastolic by antihypertensive drugs contributes to a 22% reduction in coronary heart disease events (17%–27%) and a 41% (33%–48%) reduction in stroke.^93^ Could qigong be used as an alternative therapy to antihypertensive drugs for hypertension? In our review, compared with antihypertensive drugs, qigong had a clinically meaningful but nonsignificant effect on lowering SBP but was effective in lowering DBP. Therefore, qigong may be a valuable lifestyle intervention in maintaining a desirable BP. However, due to the limited number of included trials and significant clinical heterogeneity in this subgroup, additional evidence is needed to confirm these conclusions.

TCM is often used in addition to baseline treatment with effective Western medicines to enhance the hypotensive effect and reduce the toxicity of conventional treatment. That is, patients in the control groups received conventional Western medicine therapy alone, whereas patients in the treatment group received a combination of TCM and Western medicine therapy. This design is also known as an add-on design, which is quite popular for TCM studies of various diseases and conditions.^94–99^ Is adjunctive qigong therapy more effective than antihypertensive drugs alone in lowering BP? The fourth finding was the evaluation of the effect of the combination of qigong and antihypertensive drugs on BP. Our data suggest that QPADs significantly decrease both SBP and DBP more than antihypertensive drugs alone. A similar positive effect on BP was also reported in other nonpharmacological add-on studies for hypertension.^10,12,14^ These results suggest that QPAD therapy may be an optimal therapeutic regimen for hypertensive patients who are insensitive to pharmacological treatment alone, particularly when compliance with antihypertensive drug treatment is poor. Considering the potential BP reduction effect of qigong as a monotherapy or adjunctive therapy, its practice could reduce the global burden of disease due to high BP either economically or clinically.

A recent review by Lawes et al^100^ suggested that approximately 54% of stroke and 47% of ischemic heart disease worldwide are attributable to high BP. Previous studies have also demonstrated that physical activity reduces cardiovascular mortality by 16% to 67%.^101^ Could qigong contribute to the reduction of cardiovascular events and regular exercise? We estimated the effect of qigong in preventing the incidence of all causes of mortality and progression to severe complications. Only 1 trial assessed these outcomes with a 30-year follow-up, and further evidence is required.^59^ A total of 242 hypertensive patients were randomized into a QPADs group (n = 122) and an antihypertensive drugs group (n = 120) for 12 months. At the end of follow-up, a significant beneficial effect on total mortality was observed in the QPADs group (25.41% vs 47.50%, *P* < 0.001), particularly mortality caused by serious hypertensive complications (18.85% vs 39.16%, *P* < 0.01). Although positive effects of qigong on cardiovascular morbidity and mortality were reported by only 1 trial (5%, 1/20) with long-term follow-up, reporting was inadequate in the other 19 trials (95%, 19/20), demonstrating that the long-term effect of qigong could be evaluated with RCTs and that well-designed studies with a low risk of bias generate more-valid clinical evidence.

In addition, it should be noted that doctor training and expertise are important factors contributing to the positive effects of qigong for hypertensive patients. More recent studies have suggested that the choice of treatment by doctors is influenced by their training and clinical experience.^102^ Doctors with further elective training in TCM used more individualized TCM therapies. Thus, studies conducted in China are likely completed by qigong experts with better training and experience. Is there any difference in reporting the participation of experienced physicians? In this review, 2 RCTs that were preformed in South Korea all declared the use of qigong experts.^44,45^ Another trial that was published in English also stated that qigong was taught by an instructor with expertise in *guolin* qigong.^46^ Thus, all trials conducted outside China and published in English gave full consideration to this issue, and no difference in doctor training or expertise was identified.

## LIMITATIONS

The apparent reported positive findings should be interpreted conservatively due to the low methodological quality and significant heterogeneity of the included trials. There are 5 limitations to this review. First, methodological issues are a common concern in CAM clinical trials.^103^ The results reported in this article are similar to those of previous systematic reviews,^21,22^ which also addressed concerns regarding the high risk of bias in the primary studies. In this review, most of the trials were of poor methodological quality. Inadequate reporting of the study design, allocation sequence, allocation concealment, blinding, intention-to-treat analysis, and dropouts was identified in the majority of trials. Randomization is necessary to avoid selection bias. However, only 1 trial reported the method of random sequence generation.^46^ In addition, no trial described allocation concealment, suggesting that some declared RCTs may not be true RCTs. Blinding is an essential method for preventing research outcomes from being influenced by either performance bias or detection bias. In this review, because it is difficult to blind patients to treatment, only the blinding of the outcome assessor was considered according to the Cochrane risk of bias criteria. However, no trials reported this blinding. The lack of information on dropouts and withdrawals was also problematic. Only 4 trials provided information on dropout rates and withdrawals.^44–46,59^ Moreover, a lack of follow-up might lead to difficulty in accounting for the long-term effect of qigong. Only 1 study described a pretrial estimation of the sample size.^46^ Therefore, whether the sample sizes met the basic requirements of clinical research in the other trials is unknown.

Second, the heterogeneity in this analysis merits further attention. The significant clinical heterogeneity reflected in variations in methodological quality, participants, interventions, and antihypertensive drugs might weaken the reliability of the data. Thus, it was not possible to perform a pooling analysis of the trials.

Third, potential publication bias is another major concern that would limit the generalizability of the findings. The large number of duplicate publications was also a matter of concern in this review. Notably, the Shanghai Institute of Hypertension in China has completed numerous studies including >2000 patients in the past 60 years, although few of these studies are represented in the literature. After checking the original data, only 4 of 25 published articles were included.^57–59,61^ Although great effort was made to avoid language bias and location bias during data retrieval, almost all trials identified after comprehensive searches were conducted in China and published in Chinese with positive results favoring qigong treatment. A systematic review by Vickers et al^104^ reported that some countries, including China, publish a high proportion of positive results and found publication bias to be a possible explanation. Therefore, we cannot completely rule out potential publication bias.

Fourth, although most studies claimed that antihypertensive drugs were used to achieve the best control of BP, whether these prescriptions met clinical standards was unclear due to insufficient reporting. In addition, the use of 3-grade evaluation criteria to evaluate the overall effect on BP limits the global acceptability and generalizability of these findings.

Furthermore, there was little detailed information about AEs. Appropriate qigong exercise may induce a series of normal physiological and psychological reactions in participants; however, inappropriate training might lead to physical and mental disorders.^105–107^ Qigong as evaluated in this review generally seemed safe and well tolerated by hypertensive patients. However, the safety of its use could not be confirmed because only 1 study mentioned the safety of interventions. Investigators may have underestimated possible AEs in these trials. Therefore, it is difficult to draw definite conclusions about the safety of qigong.

## CONCLUSIONS

Whether qigong is beneficial for hypertensive patients is an important question. Based on the available literature in English and Chinese, the results of this meta-analysis suggest that qigong is an effective therapy for hypertension. Qigong is superior to no intervention and antihypertensive drugs but inferior to exercise in lowering BP; qigong as an adjunctive therapy to antihypertensive drugs significantly lowers BP and could be recommended as a complementary approach for hypertensive patients. However, due to the poor methodological quality of the included studies, further rigorously designed RCTs with long-term follow-up focusing on hard clinical outcomes are required to confirm the results and to provide a high level of evidence, particularly to support qigong as an alternative to regular exercise for elderly patients. If well-designed RCTs with a high quality of methodology confirm that qigong is beneficial, it could be recommended as an evidence-based complementary and alternative therapy for the treatment of hypertension worldwide. On the contrary, negative results would challenge the rational basis and clinical evidence supporting qigong. We hope that this systematic review paves the way for evidence-based research on qigong for hypertension.
